# In vitro inhibition of platelets aggregation with generic form of clopidogrel versus branded in patients with stable angina pectoris

**DOI:** 10.15171/jcvtr.2017.33

**Published:** 2017-10-10

**Authors:** Rza Hajizadeh, Samad Ghaffari, Mojtaba Ziaee, Behrooz Shokouhi, Ahmad Separham, Parvin Sarbakhsh

**Affiliations:** ^1^Cardiovascular Research Center, Tabriz University of Medical Sciences, Tabriz, Iran; ^2^Medicinal Plant Research Center, Institute of Medicinal Plants, ACECR, Karaj, Iran

**Keywords:** Stable Angina, Clopidogrel, Generic, Brand, Platelet Aggregation

## Abstract

***Introduction:*** Clopidogrel is a potent platelet activation and aggregation inhibitor that prevents thrombosis in coronary artery diseases (CADs). In comparison to locally produced generic one (Osvix®), original brand of clopidogrel (Plavix®) is expensive. This study was designed to evaluate the effectiveness and uniformity of Osvix® versus Plavix® in patients with percutaneous coronary intervention (PCI) by means of platelet aggregation indexes.

***Methods:*** This randomized, double blind clinical study was conducted at Shahid Madani heart hospital, Tabriz, Iran, and 129 patients with previous PCI were enrolled in two independent treatment groups. All patients participated in this study were on dual antiplatelet therapy at least for 30 days. ASA 80 mg/d and clopidogrel 75 mg/d and a stat dose of 300 mg of clopidogrel before PCI were administered for all patients. To evaluate the anti-platelet activity, blood samples were taken from the patients and platelet aggregation test was performed.

***Results:*** The total study population represents a group of 129 patients (99 men and 30 women) with mean age of 57.7 ± 9.7 years with stable angina pectoris. The baseline characteristics and laboratory findings of two groups (except mean platelet volume [MPV]) were not different statistically. The mean platelets aggregation at 30th day was 13.7±7.0 in Plavix® group and 14.8±5.8 in Osvix® group (*P* value = 0.35).

***Conclusion:*** This study showed that Osvix® as a generic form of clopidogrel was not significantly different from the original brand (Plavix) in terms of in vitro platelet inhibition.

## Introduction

Ischemic heart disease (IHD) is the largest single cause of mortality in industrialized countries and is one of the top killer diseases in underdeveloped countries as well.^1-3^ The stable angina is the prototypical apparition of IHD, among many others. Platelets aggregation and blood coagulation are major risk factors result in the so-called atherothrombotic process.^4^ This phenomenon after coronary stenting could be catastrophic. Antiplatelet therapy is indicated to prevent cardiovascular events due to thrombosis in patients with stable angina/stable IHD (SIHD). Co-administration of clopidogrel plus ASA as anti-platelet therapy has been established the standard treatment for the reduction of adverse events in patients with coronary artery disease (CAD) and those undergoing percutaneous coronary intervention (PCI) to decrease the rate of morbidity and mortality.^5^ The thienopyridine derivatives such as clopidogrel are antiplatelet drugs that prevent platelet function by selective and irreversible inhibition of adenosine diphosphate (ADP) binding to P2Y12 receptor on platelets.^6^ This leads to a reduction in activity of platelet glycoproteins (GPIIb/IIIa) required for fibrinogen platelet adhesion.^7^

In recent years, prescription of clopidogrel has risen sharply due to increasing prevalence of coronary heart disease (CHD) and stenting therapy in Iran.^7,8^ Several generics versions of clopidogrel have been brought to the market in Iran. In recent years many patients and health care insurances have persuaded the prescription of generic drugs to control pharmaceutical costs, whose market share grown rapidly and surpassed 40% of the market volume in most industrialized nations.^9^ The most common generic form of clopidogrel in Iran named “Osvix^®^” has been brought by Osveh Pharmaceutical Company. Some of previous studies have showed that original clopidogrel (Plavix) and generic clopidogrel (Osvix^®^) were acceptable and similar in their pharmacokinetic and pharmacodynamic properties but other studies reported different results.^7,8,10,11^ Khosravi and colleagues reported that Plavix^®^ and Osvix^®^ did not have significant difference regarding major cardiovascular events and side-effect profile.^8^ On the other hand, Imani et al reported that Osvix^®^ was significantly different from Plavix in reduction of risk of blood coagulation in patients undergoing coronary artery bypass grafting (CABG) operation.^11^

Iranian Food and Drug Administration approved Osvix^®^ for the treatment of patients undergoing PCI and stent implantation. Some health care professionals and patients have declared concern that generic and brand-name medicines may not be equivalent in their outcomes on various clinical parameters, including physiological and important laboratory measurements such as platelet aggregation, and traces such as health system utilization or mortality.^10,12,13^

Therefore, the aim of this study was to evaluate the effectiveness of two brand preparations of clopidogrel 75 mg tablets (Osvix^®^ and Plavix^®^) in patients with stable angina/stable IHD (SIHD) by means of platelet aggregation index.

## Materials and Methods

### Study design

This study was randomized, double-blind, parallel-group conducted at Shahid Madani heart hospital, Tabriz University of Medical Sciences, Tabriz, Iran.

### Patients

A total of 129 patients aged 18 to 70 years old with stable angina (documented myocardial ischemia by angiography) who underwent PCI were enrolled eligible. All the patients underwent complete history and physical examination. Detailed baseline demographic and clinical data including sex, age, cardiac interventions were recorded according to the patients’ medical charts. History of smoking, hypercholesterolemia, hypertriglyceridemia, high blood pressure, diabetes mellitus and family history of premature CHD in first relatives were also collected. All the patients underwent electrocardiography and echocardiography before the intervention. Patients were randomly assigned into two groups: one group (68 patients) got a brand product (Plavix^®^) and the other (61 patients) received the locally manufactured Osvix^®^. Diabetic and high blood pressure patients allocated to both groups equally. The two forms were previously labeled as A and B. Both the groups received a loading dose of Plavix^®^ or Osvix^®^ (1:1), 300 mg (4 tablets of 75 mg) on day 1, followed by 75 mg/d at morning daily for the next 30 days. All patients received daily aspirin 80 mg/d during the study period, respectively. Exclusion criteria were as follows: any exercise within 15 to 20 minutes before taking blood sample, the treatment with fibrinolytic drugs, IIb/IIIa inhibitors, ticlopidine, taking warfarin, NSAID, antibiotic, antihistamines, theophylline and tricyclic antidepressant within past 2 weeks, history of allergy or intolerance or contraindication to aspirin or clopidogrel, increased risk of major bleeding: hemorrhagic diathesis, platelet count <100 ×10^9^/L, serum creatinine>1.8 mg/dL, and/or liver disease resulting in bilirubin >2 mg/dL, malignancies, body mass index (BMI) under 18 and over 30. Laboratory tests included hemoglobin, hematocrit, leukocyte count, platelet count, prothrombin time (Pt), activated partial thromboplastin time (Ptt), international normalized ratio (INR), plasma glucose measurement, serum creatinine. Also all medications taken by patients in 30 days of study were recorded. Patients provided written informed consent before inclusion in the trial.

### Methods

The pharmacy supplied medications in similar sealed opaque packs. Physicians and patients who involved in this study were blinded as to the clopidogrel brand. Patients followed up to assess the antiplatelet effect of the two different clopidogrel brands and clinical outcome. At the 30-day follow-up visit, blood samples of ~ 15 mL were drawn in a fasting state with a loose tourniquet through a short venous catheter inserted into a forearm vein. Samples collected in one-tenth volume of 3.8% sodium citrate for platelet function testing 2–4 hours after the last intake of the medications. Patients were asked to avoid high fat meal and caffeine drinks. All laboratory staff conducting the platelet aggregation assessment were blinded as to patient treatment.

Platelet aggregation was monitored the point-of-care during 2 hours after sampling. The platelet rich plasma (PRP) was stored at room temperature (22°C) prior to experiment and at 37°C 1 minute before performing the aggregation tests. Immediately after addition of ADP (5 μM) as the agonist to prepared samples platelet aggregation allowed to proceed. Evaluation of aggregation was done in siliconized tubes at 37°C in constant rotation rate of 1000 rpm and curves were recorded for 5 minutes. Aggregation was assessed as change in light transmission and reported as the maximum percentage change from baseline using platelet-poor plasma as a reference. All samples were analyzed in duplicate to ensure accuracy.

### Statistical methods

We calculated sample size based on previous studies of 66 patients per group (132 total) would give the study 90% power to detect the non-inferiority treatment.

Baseline characteristics were described using percentages for categorical variables and means ± standard deviation (SD) for quantitative variables. All measurement data were analyzed using the Kolmogorov-Smirnov test. Comparisons between the two groups were performed by means of an unpaired *t* test for continuous variables. The chi-square test was used for categorical data. Categorical data are presented as percent frequencies. *P* value of 0.05 or less was considered significant. All the statistical analyses were performed using SPSS version 23.0 (SPSS, USA).

## Results

The total study population represents a group of 129 patients (99 men and 30 women) with mean age of 57.7 ± 9.7 years with stable angina pectoris. The mean age of subjects in Plavix^®^ group and Osvix^®^ group showed no statistical difference (*P* = 0.32). Demographic characteristics and drug history of patients are shown in Table 1; Frequency of hypertension, diabetes mellitus, smoking and previous medical history were similar between Plavix^®^ and Osvix^®^ groups.

**Table 1:** Demographic characteristics of patients

		**Plavix® (n=68)**	**Osvix® (n=58)**	***P*** ** value**
Gender	Male	55 (80.9%)	44 (72.1%)	0.24
	Female	13 (19.1%)	17 (27.9%)	
Age, years	58.60±9.39	56.89±10.22	0.32
DM	Yes	17 (25.0%)	17 (27.9%)	0.71
	No	51 (75.0%)	44 (72.1%)	
HTN	Yes	33 (48.5%)	29 (47.5%)	0.91
	No	35 (51.5%)	32 (52.5%)	
Smoking	Yes	12 (17.6%)	16 (26.2%)	0.24
	No	56 (82.4%)	45 (73.8%)	
Beta blocker	Yes	55 (89.6%)	47 (77.0%)	0.59
	No	13 (19.1%)	14 (23.0%)	
ARB	Yes	36 (52.9%)	23 (37.7%)	0.08
	No	32 (47.1%)	38 (62.3%)	
Atorvastatin	Yes	59 (86.8%)	52 (86.8%)	0.80
	No	9 (13.2%)	9 (14.8%)	
Calcium blocker	Yes	14 (20.6%)	7 (11,5%)	0.16
	No	54 (79.4%)	54 (88.5%)	

The mean platelets aggregation at 30th day 2-4 hours after dosing in response to 5 μM/L ADP (*P* ≤ 0.05) was 13.7±7.0 in Plavix^®^ group and 14.8±5.8 in Osvix^®^ group (*P* = 0.35). There was no significant difference between generic and brand clopidogrel in platelet aggregation inhibitory activity. Patients’ data is shown in Table 1.

Laboratory data of patients in Plavix^®^ and Osvix^®^ groups shown in Table 2. These results show no significant difference between two medicines, except for mean platelet volume (MPV). This parameter (MPV) was significantly higher in Osvix^®^ group. (*P* = 0.006). Bleeding (major or minor) and drug reaction was not reported by patients in study groups.

**Table 2:** Laboratory data of patients

	**Plavix® (n=68)**	**Osvix® (n=58)**	***P *** ** value**
WBC	6718.24±1585.17	6298.52±1661.42	0.15
Hb	14.30±1.49	13,88±1.36	0.09
Neutrophil	3925.15±1150.54	3540.98±1501.98	0.10
Basophil	55.74±30.73	50.98±24.54	0.34
Monocyte	398.97±124.16	377.70±119.67	0.33
Lymphocyte	1964.71±572.11	2013.93±525.85	0.61
PLT	228.41±95.15	221.30±51.57	0.60
MPV	9.31±1.59	9.98±1.13	0.006
FBS	123.40±43.32	144.82±108.92	0.16
PT	1256±0.48	12.70±0.66	0.18
PTT	34.59±4.27	33.70±4.85	0.27
INR	1.04±0.08	1.05±0.09	0.26
Creatinine	1.11±0.27	1.08±0.20	0.42

## Discussion

Clinicians are frequently encounter with anecdotal evidence from various sources such as patients, physicians or pharmaceutical representatives asserting that generic drugs are not effective enough or has more side effects in comparison to their branded rivals. Multiple investigations show satisfying outcomes with generics; however their use is still prudent in various states. Anti-platelets drugs dominate the cardiovascular pharmaceutical market.

Platelets play a pivotal role in hemostasis and the progress of arterial thrombosis.^4,14^ Platelets adhesion, activation and aggregation has major role in cardiovascular complications in stable angina patients.^4,15^ Antiplatelet drugs such as aspirin and clopidogrel reduce the rate of cardiovascular events and hence reduce mortality and morbidity in patients with CAD in early and long time.^16,17^ Clopidogrel is an oral, thienopyridine-class antiplatelet agent that inhibits ADP receptor-mediated platelet activation.^8,18,19^ For years Plavix^®^ (clopidogrel) was widely prescribed and became the second best-selling drug in the world.^20^ Economic difficulties and prohibitive costs make barrier to access the necessary medicines.^21^ Locally provided clopidogrel can increase the access with affordable prices. When a generic drug comes to the market, healthcare providers and consumers have worried about safety, efficacy and clinical outcomes such as health system utilization or mortality of new generic in comparison to brand-name ones.^22^ Several studies such as OPCES have compared various aspects of Plavix^®^ and Osvix^®^. These observations showed different results regarding therapeutic and adverse effects between two different products.^8,10,23^

The objective of the present study was to compare the changes in platelet aggregation induced by 5 μmol/L ADP 30 days after administration of clopidogrel. The data from this study indicated a significant inhibition of ADP-induced platelet aggregation after clopidogrel administration. We observed that a loading dose of 300 mg followed by 75 mg/d of both brand of clopidogrel inhibit of platelet aggregation similarly.

Patients monitored for episodes of angina, and adverse drug reactions in the initial hospital period and following admissions. None of patients developed angina pectoris or drug reactions during 30 days follow up. Khosravi et al studied 442 patients with chronic stable angina. In this study mid-term mortality and the incidence of major adverse events in Osvix^®^ and Plavix^®^ groups statistically were not different (*P* = 0.61 and 0.26 respectively)^8^; our study supports their results. Imani et al reported different results. They studied 80 patients undergoing coronary bypass grafting. Patients divided to Plavix and Osvix^®^ groups. Their results showed a significant difference in the rate of platelet-rich plasma between two groups. The main limitation of their study was sending blood samples to other city (Tehran) in different intervals and their smaller sample size.^11^ All of samples in our investigation were analyzed during 2 hours after blood samples were obtained.

Recent investigations have evaluated the association of White blood cell (WBC) count, neutrophil count, and neutrophils/lymphocytes rate as an independent prognostic predictor both in acute myocardial infarction and in stable angina.^24^ Lymphocytes also may have an essential role in modulating the inflammatory response in atherosclerotic process and comparative lymphopenia in myocardial infarction was pretended as a stress reflex conducted by elevated plasma cortisol.^25^ Our results showed no significant difference in demographic and our laboratory results despite MPV. This parameter was significantly higher in Osvix^®^ group. MPV, is a useful predictor of platelets activity and associated with an increased risk of acute myocardial infarction (AMI) and other cardiovascular events.^26^ Higher levels of MPV is associated with a worse outcomes in cardiovascular events such as restenosis following coronary angioplasty and acute cerebrovascular events.^26,27^

Similar to our results Khosravi et al reported no difference in neutrophil and platelet count among two groups.^8^

The effect of generic and branded clopidogrel on thrombin generation such as activated partial thromboplastin time (aPTT), prothrombin time/INR, were regularly performed for all subjects to evaluate bleeding and coagulation dysfunctions in period of medical examinations which showed no difference.

Further research is needed to determine whether MPV provides excessive diagnostic value in being able to notice patients at elevated clinical risk and whether medical care modification of this marker may lead to improved cardiovascular care.

## Ethical issues

The study protocol was approved by the institutional ethics committee of Tabriz University of medical sciences. The study was carried out in compliance with the principles of the Declaration of Helsinki and its amendments. All patients provided written informed consents for participation.

## Competing interests

All authors declare no competing financial interests exist.

## Acknowledgments

Our investigation was supported by the grant from Cardiovascular Research Center of Tabriz University of Medical Sciences, Tabriz, Iran.

## References

[1] Lozano R, Naghavi M, Foreman K, Lim S, Shibuya K, Aboyans V (2013). Global and regional mortality from 235 causes of death for 20 age groups in 1990 and 2010: a systematic analysis for the Global Burden of Disease Study 2010. Lancet.

[2] Ziaee M, Khorrami A, Nourafcan H, Ebrahimi M, Amiraslanzadeh M, Garjani M (2015). Cardioprotective effects of essential oil of Lavandula angustifolia on isoproterenol-induced acute myocardial infarction in rat. Iran J Pharm Res.

[3] Moran AE, Forouzanfar MH, Roth G, Mensah GA, Ezzati M, Flaxman A (2014). The global burden of ischemic heart disease in 1990 and 2010: the Global Burden of Disease 2010 study. Circulation.

[4] Badimon L, Padró T, Vilahur G (2012). Atherosclerosis, platelets and thrombosis in acute ischaemic heart disease. Eur Heart J.

[5] Jernberg T, Payne CD, Winters KJ, Darstein C, Brandt JT, Jakubowski JA (2006). Prasugrel achieves greater inhibition of platelet aggregation and a lower rate of non-responders compared with clopidogrel in aspirin-treated patients with stable coronary artery disease. Eur Heart J.

[6] Tsoumani ME, Kalantzi KI, Goudevenos IA, Tselepis AD (2014). Clopidogrel generic formulations in the era of new antiplatelets: a systematic review. Curr Vasc Pharmacol.

[7] HajiAghajani M, Kobarfard F, Safi O, Sheibani K, Sistanizad M (2013). Resistance to Clopidogrel among Iranian Patients Undergoing Angioplasty Intervention. Iran J Pharm Res.

[8] Khosravi AR, Pourmoghadas M, Ostovan M, Mehr GK, Gharipour M, Zakeri H (2011). The impact of generic form of Clopidogrel on cardiovascular events in patients with coronary artery stent: results of the OPCES study. J Res Med Sci.

[9] Manzoli L, Flacco ME, Boccia S, D’Andrea E, Panic N, Marzuillo C (2016). Generic versus brand-name drugs used in cardiovascular diseases. Eur J Epidemiol.

[10] Souri E, Jalalizadeh H, Kebriaee‐Zadeh A, Shekarchi M, Dalvandi A (2006). Validated HPLC method for determination of carboxylic acid metabolite of clopidogrel in human plasma and its application to a pharmacokinetic study. Biomed Chromatogr.

[11] Imani B, Safi-Ariyan R, Manaafi B, Karampourian A, Ghazikhanlou Sani K (2014). The effectiveness, side effects and acceptability of locally available brand of Clopidogrel (Osvix) as antiplatelet tablet in CABG patients. Tehran Univ Med J.

[12] Shrank WH, Liberman JN, Fischer MA, Girdish C, Brennan TA, Choudhry NK (2011). Physician perceptions about generic drugs. Ann Pharmacother.

[13] Hassali MA, Shafie AA, Jamshed S, Ibrahim MI, Awaisu A (2009). Consumers’ views on generic medicines: a review of the literature. Int J Pharm Pract.

[14] Burnouf T, Goubran HA, Chou M-L, Devos D, Radosevic M (2014). Platelet microparticles: detection and assessment of their paradoxical functional roles in disease and regenerative medicine. Blood Rev.

[15] Brydon L, Magid K, Steptoe A (2006). Platelets, coronary heart disease, and stress. Brain Behav Immun.

[16] 
Capodanno
 
D
, 
Angiolillo
 
DJ
 (2013). Management of antiplatelet therapy in patients with coronary artery disease requiring cardiac and noncardiac surgery. Circulation.

[17] Dewilde WJ, Oirbans T, Verheugt FW, Kelder JC, De Smet BJ, Herrman J-P (2013). Use of clopidogrel with or without aspirin in patients taking oral anticoagulant therapy and undergoing percutaneous coronary intervention: an open-label, randomised, controlled trial. Lancet.

[18] Yusuf S, Zhao F, Mehta SR, Chrolavicius S, Tognoni G, Fox KK, Clopidogrel in Unstable Angina to Prevent Recurrent Events Trial Investigators (2001). Effects of clopidogrel in addition to aspirin in patients with acute coronary syndromes without ST-segment elevation. N Engl J Med.

[19] Bertrand ME, Rupprecht H-J, Urban P, Gershlick AH (2000). Double-blind study of the safety of clopidogrel with and without a loading dose in combination with aspirin compared with ticlopidine in combination with aspirin after coronary stenting the clopidogrel aspirin stent international cooperative study (CLASSICS). Circulation.

[20] Choudhry NK, Levin R, and Avorn J (2008). The economic consequences of non–evidence-based clopidogrel use. Am Heart J.

[21] Brandt JT, Payne CD, Wiviott SD, Weerakkody G, Farid NA, Small DS (2007). A comparison of prasugrel and clopidogrel loading doses on platelet function: magnitude of platelet inhibition is related to active metabolite formation. Am Heart J.

[22] Kesselheim AS (2011). Off-label drug use and promotion: balancing public health goals and commercial speech. Am J Law Med.

[23] Khosravi AR, Raoufi A, Pourmoghadas M, Paydari N, Gharipour M, Namdari M (2013). Late clinical events of drug eluting versus bare metal stenting; OPCES’ancillary study. Pak J Med Sci.

[24] Madjid M, Awan I, Willerson JT, Casscells SW (2004). Leukocyte count and coronary heart disease: implications for risk assessment. J Am Coll Cardiol.

[25] Şahin DY, Elbasan Z, Gür M, Yıldız A, Akpınar O, Icen YK (2013). Neutrophil to lymphocyte ratio is associated with the severity of coronary artery disease in patients with ST-segment elevation myocardial infarction. Angiology.

[26] Chu S, Becker R, Berger P, Bhatt D, Eikelboom J, Konkle B (2010). Mean platelet volume as a predictor of cardiovascular risk: a systematic review and meta‐analysis. J Thromb Haemost.

[27] Greisenegger S, Endler G, Hsieh K, Tentschert S, Mannhalter C, Lalouschek W (2004). Is elevated mean platelet volume associated with a worse outcome in patients with acute ischemic cerebrovascular events?. Stroke.

